# How to maximize the therapeutic effect of exosomes on skin wounds in diabetes mellitus: Review and discussion

**DOI:** 10.3389/fendo.2023.1146991

**Published:** 2023-03-27

**Authors:** Jia Dong, Bin Wu, Weidong Tian

**Affiliations:** ^1^ Department of Stomatology, People's Hospital of Longhua Shenzhen, Shenzhen, Guangdong, China; ^2^ State Key Laboratory of Oral Disease & National Clinical Research Center for Oral Diseases & National Engineering Laboratory for Oral Regenerative Medicine, West China School of Stomatology, Sichuan University, Chengdu, Sichuan, China

**Keywords:** diabetes, skin wounds, exosomes, maximize, therapeutic effect

## Abstract

Chronic skin wound healing, especially in diabetes mellitus, is still unsolved. Although many efforts have been made to treat diabetic skin wounds, current strategies have achieved limited effectiveness. Nowadays, a great number of studies have shown that exosomes might be a promising approach for treating diabetic wounds. Many studies and reviews have focused on investigating and discussing the effectiveness and mechanism of exosomes. However, maximizing its value in treating skin wounds in diabetes mellitus requires further consideration. In this review, we reviewed and discussed the aspects that could be further improved in this process, including finding a better source of exosomes, engineering exosomes, adjusting dosage and frequency, and combining more efficient delivery methods. This review provided an overview and idea of what we can do to improve the therapeutic effect of exosomes on skin wounds in diabetes mellitus. Only by combining all the factors that affect the effectiveness of exosomes in diabetic wound healing can we further promote their clinical usefulness.

## 1 Introduction

The healing of chronic skin wounds, especially diabetic skin wounds, is one of the most intractable problems for clinicians and a heavy burden for patients, both physically and financially (1, 2). To date, there are numerous strategies and methods to treat diabetic wounds, and however, these are not exempt from limitations (3, 4). Hence, there is a crucial and urgent need for effective and safe methods to promote diabetic wound healing. Exosomes are one type of extracellular vesicles (EVs) secreted by various cells and show a double-layer membrane structure and a particle size ranging from 30 to 200 nm. They are involved in cell-cell communication and intracellular signaling. Exosomes show a lot of advantages, such as being stable, easily stored, and not rejected by the immune system, offering a homing effect, and the dosage can be easily controlled (5). Recent research results indicated that exosomes participated in the development and outcome of diabetes and its related complications (6). For the difficult-to-heal skin wounds caused by diabetes, exogenous exosome therapy could promote the functional recovery of multiple essential cells. They effectively promoted angiogenesis, collagen synthesis, and modulating inflammation (7), so exosome therapy might become very important in wound healing strategies in order to enhance antimicrobial stewardship (8).

Although exosome therapies hold great potential for facilitating diabetic skin wound healing and regeneration, for any drug, its clinical efficacy will also depend on many other factors, like dosage, application frequency, and delivery methods (9, 10). In this review, according to published studies on the application of exosomes in diabetic wound healing, we first summarized the characterization of skin wounds in diabetes mellitus and the role of exosomes in promoting this type of wound healing. More importantly, we reviewed and discussed the aspects that could be further improved to maximize the value of exosomes in detail.

## 2 Characterization of skin wounds in diabetes mellitus

The healing of skin wounds follows four steps, hemostasis, inflammation, proliferation, and remodeling. However, in diabetes mellitus, several factors impair these processes, making healing longer and more difficult. For example, the high glucose in diabetic wounds can lead to the gathering of bacteria and the weakening of leukocyte phagocytosis, ultimately leading to serious local infection and inflammation. The neurons are the most sensitive and initially affected cells in diabetes mellitus, and diabetic neuropathy is one of the major causes of diabetic ulcers (11). Moreover, the blood vessels of diabetic wounds are damaged, and their angiogenic capacity is weak, leading to insufficient nutrition supply and low oxygen concentration in diabetic wounds (12). In addition to an inadequate oxygen supply, high oxygen consumption by wound cells during inflammation also induces hypoxia. Likewise, hypoxia further amplifies the inflammatory response, thereby prolonging injury by increasing the levels of oxygen radicals (13). Therefore, improving these factors is the key to treating skin wounds in diabetes mellitus.

## 3 Effect of exosomes on promoting skin wound healing in diabetes mellitus

**Figure 1:**
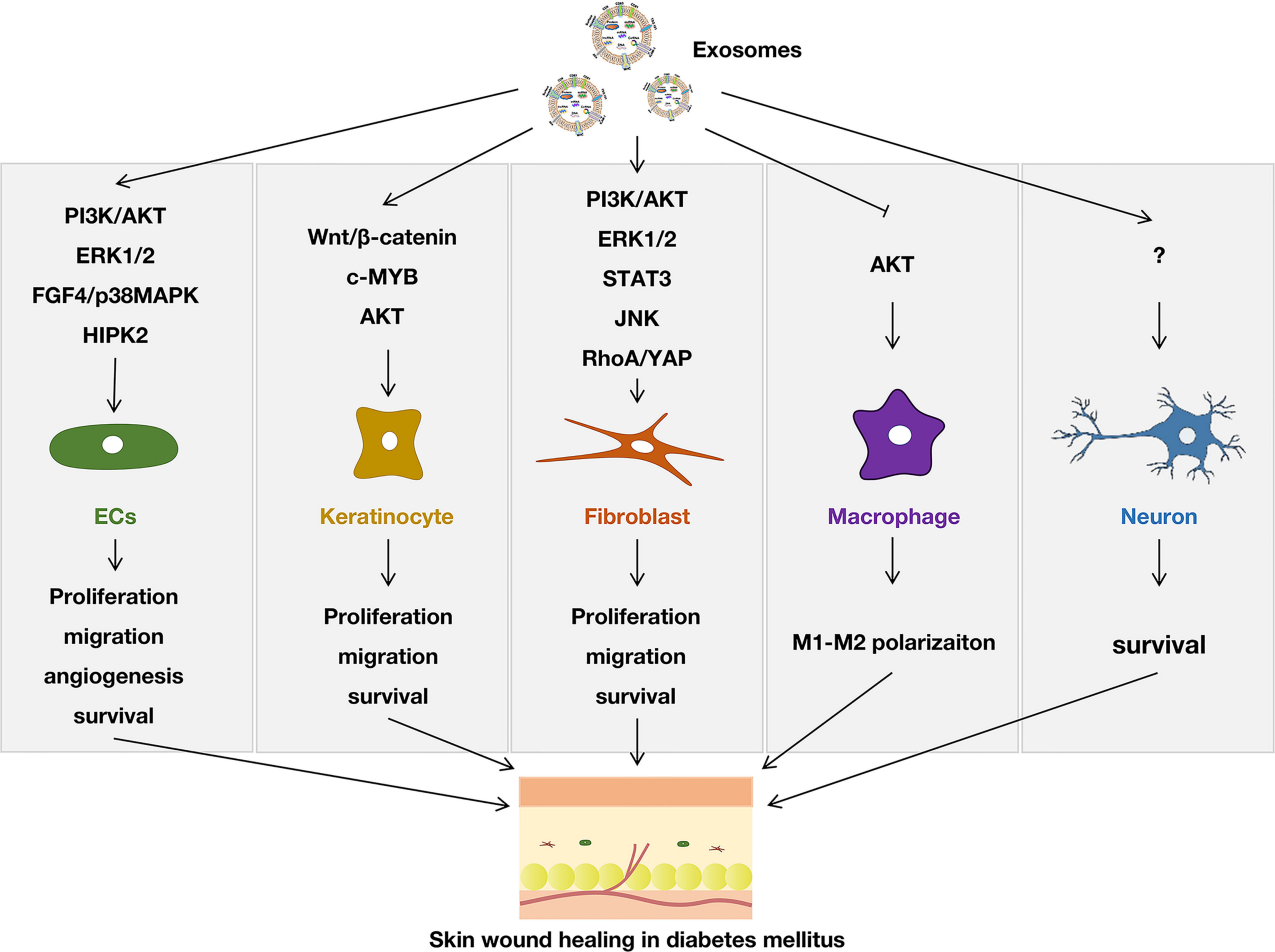
Effect of exosomes on modulating different cells in diabetic skin wound healing.

It has been reported that endothelial cells (ECs), fibroblasts, macrophages, and keratinocytes participate in angiogenesis, collagen synthesis, and anti-inflammatory processes, which are significant in diabetic wound healing. However, in the diabetic environment, the number and function of these cells are restricted to varying degrees, and the wound-healing process is delayed or interrupted. Studies found that exosomes could greatly promote survival and inhibit the apoptosis of ECs (14–17), fibroblasts (11), keratocytes (11), and neurons (11). Mostly, exosomes were reported to promote the proliferation, migration, and angiogenesis of endothelial cells, and a variety of pathways were involved, like PI3K/AKT pathway, ERK1/2 pathway, FGF4/p38MAPK pathway and HIPK2 pathway (15, 17–29). This greatly enhances the ability of local vascular regeneration in diabetic wound healing. Exosomes could also promote the proliferation and migration of keratinocytes (28, 30, 31) and fibroblasts (20, 25, 28, 32–35), and the associated pathway could be seen in **Figure 1**. It was reported that exosomes played a role in polarizing pro-inflammatory M1 macrophages to anti-inflammatory M2 macrophages (36–38), and the inhabitation of the phosphorylation of AKT might contribute to this progress (37). Especially, the increase of nerve fiber density and the functional recovery of neurons induced by exosomes played an important role in diabetic skin wound healing (39). All the functions of exosomes confirmed the potential application value of exosomes for wound healing in diabetes mellitus. However, how to maximize or further improve the therapeutic effect of exosomes is still a problem that needs a further breakthrough.

## 4 How to maximize the therapeutic effect of exosomes

### 4.1 Which is the better source of exosomes

Up to now, exosomes from various cell sources have been used to promote wound healing in diabetes. Stem cells are the most studied, including adipose stem cells (ADSCs) (11, 17, 28, 31, 35, 38, 40–47), bone marrow mesenchymal stem cells (BMSCs) (15, 16, 23, 26, 32, 33, 37, 44, 48–51), human umbilical cord-derived mesenchymal stem cells (hUCMSCs) (27, 52, 53), synovium mesenchymal stem cells (SMSCs) (18, 21), gingival mesenchymal stem cells (GMSCs) (39), human urine-derived stem cells (USCs) (22), menstrual blood-derived mesenchymal stem cells (MenSCs) (36), placental mesenchymal stem cells (PMSCs) (54), human endometrial stem cells (hEnSCs) (34), hair follicle-derived mesenchymal stromal cells (55), epidermal stem cells (ESCs) (56, 57). Other cells, like fibrocytes (58), human umbilical cord blood endothelial progenitor cells (19, 59), human umbilical cord blood mononuclear cells (hUCBMNCs) (30), macrophages (24), human amniotic epithelial cells (25), dermal fibroblasts (DFs) (14), M2 macrophages (60), human umbilical vein endothelial cells (HUVECs) (61), also were used for exosome isolation. Among these sources, ADSCs and BMSCs were chosen by most researchers. Considering the abundant sources and guaranteed effectiveness, these two kinds of stem cells might be the most reliable source of exosomes. It was reported that there were differences in the efficacy of the two types of stem cell-derived exosomes. For example, M. Pomatto et al. found that BMSCs-derived exosomes were shown to mainly promote cell proliferation, whereas ADSCs-derived exosomes demonstrated a major effect on angiogenesis (44). Therefore, the better source of exosomes still needs more comprehensive assessments.

Of course, the source of exosomes might not be limited to cells. For example, Chen et al. found that serum exosomes could accelerate diabetic wound healing by promoting angiogenesis and extracellular matrix formation (62). Guo et al. and Xu et al. isolated exosomes from platelet-rich plasma (PRP) and found this type of exosome could effectively induce the proliferation and migration of endothelial cells and fibroblasts to improve angiogenesis and re-epithelialization in diabetic skin wounds (20, 63). Besides, milk was also reported to be a source of exosome isolation (64). In our view, serum, PRP, and milk were abundant sources of exosomes. However, due to insufficient studies, its effectiveness and stability need to be further confirmed. Especially, a recent study reported that plant-derived exosomes were of therapeutic value (65); although it was not applied to skin wounds, it provided an excellent idea for exosome isolation. Perhaps it is a new direction for us to find exosomes from the proper kind of plants because many drugs contain plant extracts.

### 4.2 Engineering exosomes

**Figure 2:**
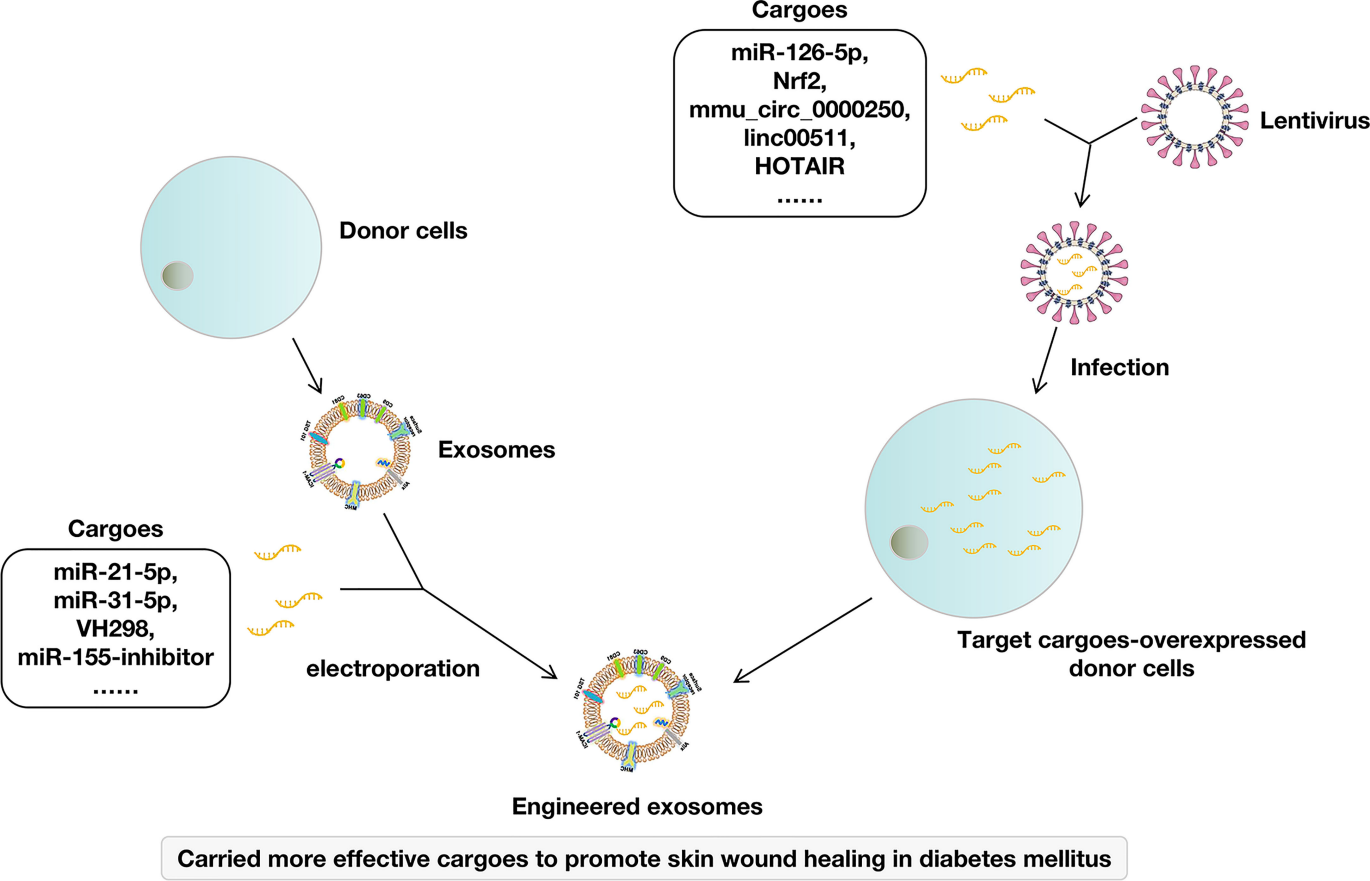
Engineering exosomes for skin wound healing in diabetes mellitus.

In addition to finding more effective natural exosomes, due to their structural characteristics, exosomes are also highly engineerable. Engineering of exosomal surface confers cell and tissue specificity. Besides, exosomes are considered delivery vehicles of diverse biological molecules, including the delivery of nucleic acid, proteins, and lipids. Studies showed that some molecules in the exosomes were particularly beneficial to wound healing, so increasing these components in exosomes through various engineering technologies could enhance the function of exosomes. Engineering strategy could be divided into direct engineering of exosomes (chemical modification and physical modification) and indirect engineering of exosomes (genetic modification of exosome-donor cells) (66). To enhance the therapeutic effect of exosomes in diabetic wound healing, some researchers have tried to use an engineering strategy (**Figure 2**).

Directly loading cargoes to exosomes was adopted by many studies. For example, direct loading miR-21-5p to ADSCs-derived exosomes by electroporation exhibited excellent effects on promoting the proliferation and migration of keratinocytes and accelerating diabetic wound healing by increasing re-epithelialization, collagen remodeling, angiogenesis, and vessel maturation (31). Loading miR-155 inhibitor to BMSCs-derived exosomes showed synergistic effects in keratinocyte migration and anti-inflammatory action, leading to accelerated wound healing by negative regulation of miR-155 (50). ESCs-derived exosomes loaded with VH298 were also found to have a better therapeutic effect on wound healing and angiogenesis in diabetes mellitus (56). Yan et al. used milk-derived exosomes as a novel system for miR-31-5p delivery and successfully encapsulated miR-31-5p mimics into milk exosomes through electroporation. Then, they proved that the miR-31-5p loaded in exosomes achieved higher cell uptake and improved endothelial cell functions *in vitro*, promoting angiogenesis and enhanced skin wound healing *in vivo* (64).

Genetic modification of donor cells was also adopted because it was a convenient and stable method. Briefly, donor cells were infected by lentivirus carrying target cargoes and stably expressed these cargoes. Then, target cargoes-carried exosomes were isolated from these donor cells. For example, SMSCs were infected by lentivirus carrying miR-126-5p. Then, miR-126-3p overexpressed exosomes (SMSCs-126-Exos) were isolated. SMSCs-126-Exos showed more effectiveness in promoting the proliferation of endothelial cells and fibroblasts and more effective in promoting angiogenesis in diabetic wound healing (18, 21). Similarly, exosomes isolated from Nrf2-overexpressed ADSCs could increase the granulation tissue formation and the levels of growth factor expression and reduce the levels of inflammation and oxidative stress-related proteins (40). Exosomes derived from mmu_circ_0000250-overexpressed ADSCs enhanced the therapeutic effect of exosomes to promote wound healing in diabetes by absorption of miR-128-3p and upregulation of sirtuin (SIRT)1 (43). Exosomes from linc00511-overexpressed ADSCs accelerated angiogenesis in diabetic foot ulcer healing by suppressing PAQR3-induced Twist1 degradation (45). Long non-coding RNA HOX transcript antisense RNA (HOTAIR)-overexpressed BMSCs produce exosomes with increased HOTAIR content that promote angiogenesis and wound healing in diabetes (48). Exosomes from mmu_circ_0001052-overexpressed ADSCs promote angiogenesis of DFU *via* miR-106a-5p and FGF4/p38MAPK pathway (17).

Other studies have enhanced the role of exosomes by changing the cultural environment of donor cells. Although it was not targeted to modify certain cargoes, it did change the cargoes in exosomes, thereby enhancing the role of exosomes in promoting diabetic wound healing. For example, Melatonin-pretreated MSCs-derived exosomes increased the ratio of M2 polarization to M1 polarization by upregulating the expression of PTEN and inhibiting the phosphorylation of AKT in diabetic wound healing (37). Exosomes derived from atorvastatin-pretreated MSCs accelerate diabetic wound repair by enhancing angiogenesis *via* AKT/eNOS pathway (26). Exosomes derived from pioglitazone-pretreated MSCs accelerate diabetic wound healing by enhancing angiogenesis (15). Hypoxia adipose stem cell-derived exosomes promote high-quality healing of diabetic wounds involving activation of PI3K/Akt pathways (35) and to improve wound healing in diabetic mice *via* delivery of circ-Snhg11 and induction of M2-like macrophage polarization (38).

Although exosome engineering used by different studies showed benefits to the therapeutic effect of exosomes, the contents of exosomes were diverse and complex. Therefore, avoiding ineffective and even harmful ingredients being transferred to the wound is difficult. In our view, the ultimate goal of exosome engineering might be to maximize the valuable components and minimize the useless components rather than focus on a single component. In addition, exosomes mainly play a role in regulating the function of cells and show no direct antibacterial effect. Therefore, it may be an effective strategy to increase its antibacterial ability to use engineering technology to wrap antibacterial drugs in exosomes.

### 4.3 Adjust the dosage and frequency

The dosage and frequency are unavoidable issues for any drug use. Some studies show that a better therapeutic effect can be achieved simply by increasing the dosage of exosomes, especially in some *in vitro* studies. For example, the proliferation and migration of fibroblasts induced by exosomes could be increased by increasing the dose of exosomes (21, 24, 32, 46). The uptake of exosomes by endothelial cells also resulted in dose-dependent increases in tube formation and angiogenesis (19, 32). For the frequency, Helena et al. reported that multiple carefully timed applications of exosomes had superior regeneration than a single dose of the same total concentration of exosomes (30). Although there are few exploratory experiments and discussions on dosage and frequency up to now, they are critical factors in the process of exosome application. They should be discussed together with the content of active cargoes in the exosomes. Therefore, it is necessary to test the dose and frequency in the application of exosomes in the same way as conventional drugs are tested (minimum effective dosage, therapeutic dosage, maximum dosage, lethal dosage, etc.).

### 4.4 Improve the delivery methods

**Figure 3:**
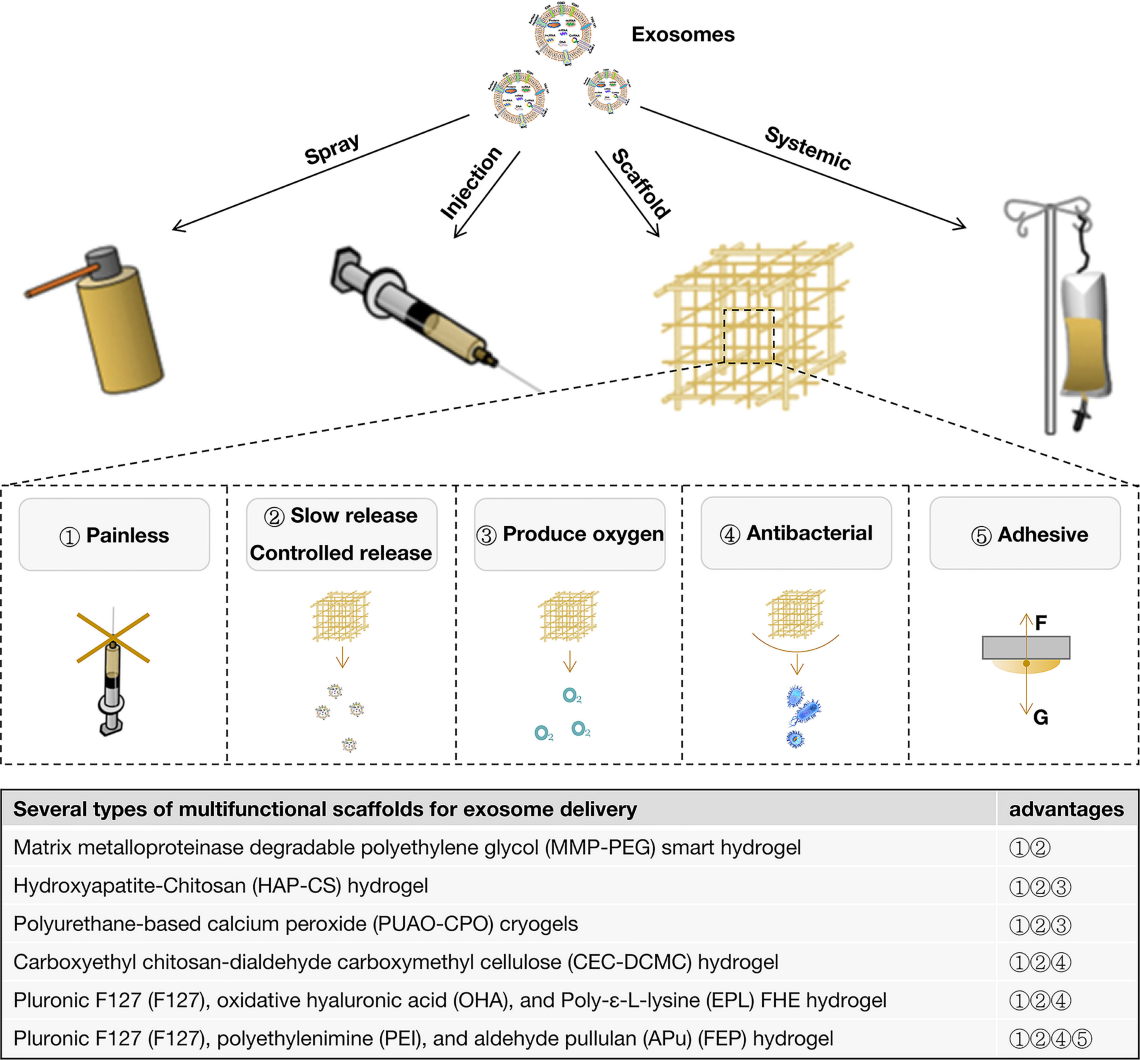
Exosome delivery methods for diabetic wounds and the advantages of scaffolds.

Through the above aspects, exosomes could solve some problems of diabetic wounds that are difficult to heal, such as difficulties in angiogenesis, nerve damage, and some inflammation problems. However, the hypoxia, bacteria, and high glucose levels have not been resolved. This requires better delivery methods to assist the therapeutic effect of exosomes. Drug delivery methods for treating skin wounds can be divided into four types: spraying, local injection, application combined with scaffold materials, and systemic application (**Figure 3**).

#### 4.4.1 Local application

Most studies delivered exosomes by subcutaneous injection around the wounds at 2 points (62), at 4 points (14, 17, 19, 22, 23, 35, 38, 43, 51, 56, 59), at multiple points (15, 26, 37) or points unknown (24, 47, 48, 50). Others combined subcutaneous injection around the wounds and injection onto the wound bed to deliver exosomes for diabetic wound healing (58). Moreover, some studies indicated that exosomes were delivered by injection onto the wound bed only (31, 57). Some studies applied exosomes by intradermal injection around the wounds (33, 36), which was regarded as a drug delivery method that could directly stimulate the active cells in the dermis. No matter which injection method was used, it can only maximize the function of exosomes themselves.

To assist the therapeutic effect of exosomes, diverse scaffolds were used to deliver exosomes. Different scaffolds played different regulatory and auxiliary roles in the function of exosomes. In general, the use of all scaffolds reduces the iatrogenic trauma and pain caused by the local injection. It has the effect of slowly releasing exosomes to varying degrees, including some simple (20, 21, 44), thermosensitive (52), photosensitive (56, 61), pH-responsive (41), and biomimetic (67) scaffolds. To control the release of exosomes, Jiang et al. fabricated a matrix metalloproteinase degradable polyethylene glycol (MMP-PEG) smart hydrogel, which could release exosomes by reacting to MMP stimulating (28). Both slow and controlled releases are designed to prolong the exosome’s action time and maintain the wounds’ local drug concentration.

Many studies improved the performance of the scaffolds, including increasing the release of oxygen (11, 18, 60) and improving the antibacterial (41, 49) and adhesive properties (42), which were important in diabetic wound healing (**Figure 3**). Hydroxyapatite (HAP) was reported to release oxygen (68, 69). Therefore, Li et al. combined HAP and Chitosan (HAP-CS) to form a hydrogel loaded with exosomes to enhance bioactivities, support angiogenesis and promote diabetic wound healing (18). Parvaiz et al. fabricated polyurethane-based oxygen-releasing antioxidant scaffolds (PUAO-CPO) to load exosomes by incorporating calcium peroxide (CPO) in polyurethane (PUAO) cryogels, which showed the sustained release of oxygen and exosomes for more than 10 days. This exosome-loaded scaffold could increase cell survival under hypoxic conditions (11). It was also reported that manganese dioxide (MnO_2_) could induce the decomposition of endogenous ROS (H_2_O_2_) into oxygen and effectively ameliorate oxidative stress and a hypoxic environment. Thus, integrating MnO_2_ into antibacterial injectable hydrogels fulfill multiple requirements, such as ROS depletion, oxygen production, and antibacterial property. Therefore, loading exosomes to this scaffold is helpful for the repair of diabetic skin wounds (60). Geng et al. indicated that carboxyethyl chitosan-dialdehyde carboxymethyl cellulose (CEC-DCMC) hydrogel showed excellent antibacterial properties and provided a physical and chemical barrier for further infection of diabetes wounds, which played an auxiliary role in the function of exosomes (49). Wang et al. also developed an injectable self-healing polypeptide-based hydrogel that exhibited inherent antibacterial activity (41). In addition to the above characteristics, some studies included the viscosity of scaffold materials to achieve good adhesion to wounds (42).

To maximize the therapeutic effect of exosomes, Wang Min et al. fabricated a thermosensitive, injectable, self-healing, and adhesive polysaccharide-based multifunctional hydrogel scaffold that exhibited efficient antibacterial activity, fast hemostatic ability, good UV-shielding performance, and pH-responsive exosome release for promoting diabetic wounds. These biomedical functions for exosomes-loaded FEP dressing probably enhance their high capability in angiogenesis and wound healing (42). Although this kind of scaffold material with extremely rich functions shows various excellent properties, it is difficult to avoid adding more complex non-medical components, which will delay its clinical transformation. Therefore, how to achieve the balance between effectiveness and safety might need to be comprehensively evaluated.

#### 4.4.2 Systemic application

For diabetic wound healing, we only found one study that delivered exosomes by systemic application *via* tail vein injection (16). For non-diabetic wound healing, one study has compared the effect of exosomes on wounds by topical injection and intravenous injection and interestingly found that intravenous injection of exosomes could enhance the healing of skin wounds compared to local injection (70). In another study, Zhou et al. systematically compared the effect of different exosome delivery methods for non-diabetic wound healing, and the results showed that the combined application of local smearing and intravenous administration offered the optimal impact on promoting wound healing, accelerating re-epithelialization, reducing scar widths, and enhancing angiogenesis and collagen synthesis (71).

**Figure 4:**
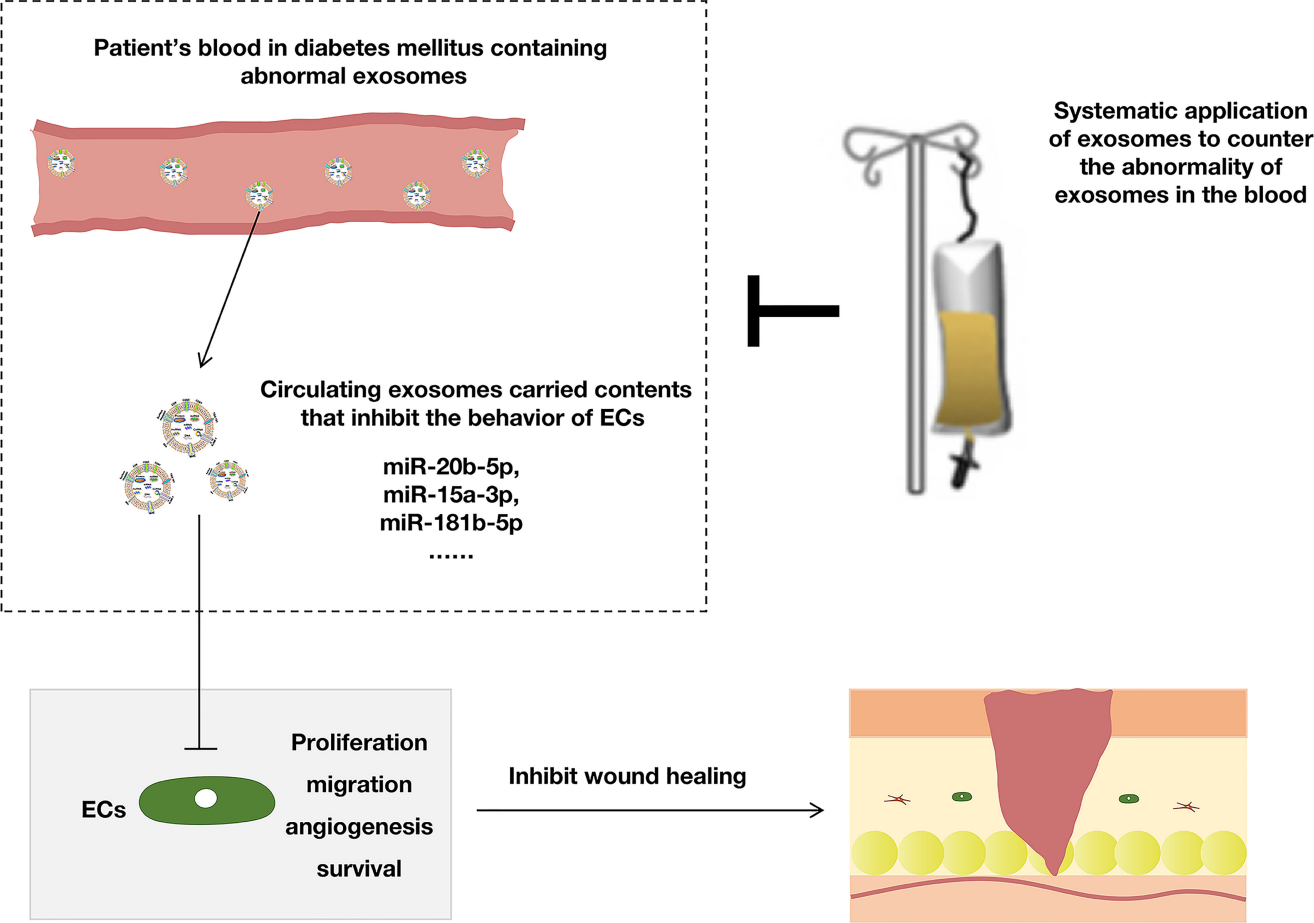
The systemic application of exosomes was beneficial to diabetic wounds.

Although the local application can play a good role in treating diabetes wounds, diabetes, as a metabolic-disorder disease, not only causes skin wounds to be difficult to heal but also faces some other physical problems, such as kidney disease, retinopathy, and neuropathy. Moreover, some studies reported that the significant upregulation of miRNAs (miR-20b-5p (72, 73), miR-15a-3p (74), miR-181b-5p (75) were observed in exosomes isolated from patients with diabetes mellitus), and these miRNAs could suppress the angiogenesis of ECs *via* different signaling pathways. Inhibition of circulating exosomal miRNAs accelerates diabetic wound repair (**Figure 4**). Therefore, we speculated that the combined local and systemic application of exosomes might benefit diabetic wound healing, and this requires further research.

## 5 Conclusions

**Figure 5:**
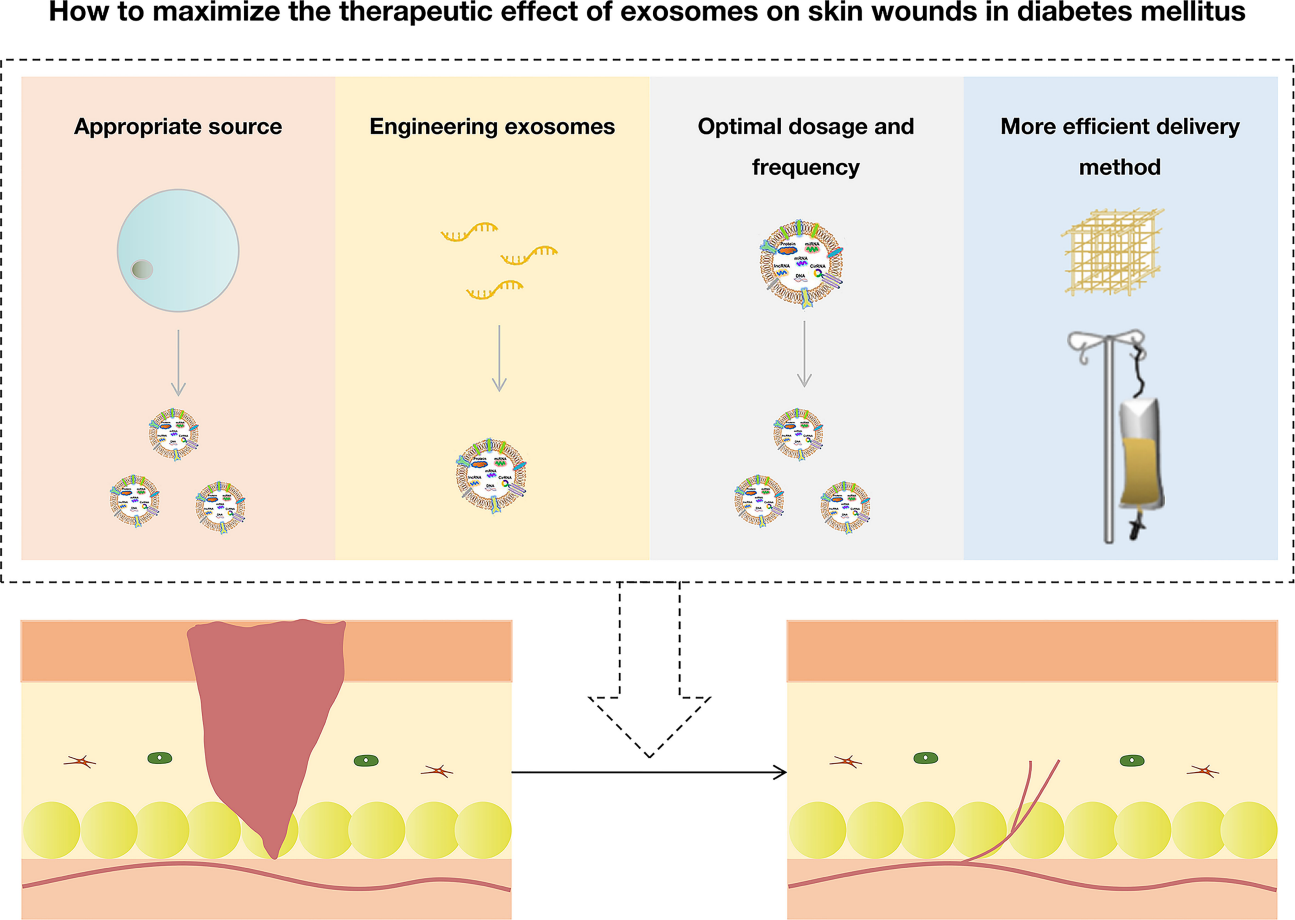
How to maximize the therapeutic effect of exosomes on skin wounds in diabetes mellitus.

Exosomes are a promising therapy for wounds in diabetes, and various ways to maximize their value are discussed in this paper. In this article, we reviewed and discussed the aspects that could be improved, including choosing appropriate donor cells, engineering exosomes, mediating dosage and frequency, and combining more efficient delivery methods (**Figure 5**). This review might provide an overview and idea for better-using exosomes to treat skin wounds in diabetes mellitus. Further reviews will be necessary to stay up to date with this rapidly evolving area of research.

## Author contributions

JD, BW, and WT jointly conceived and discussed the manuscript. JD wrote the original manuscript. WT revised the manuscript. All authors contributed to the article and approved the submitted version.
